# Technical Note: Ultra high‐resolution radiotracer‐specific digital pet brain phantoms based on the BigBrain atlas

**DOI:** 10.1002/mp.14218

**Published:** 2020-06-01

**Authors:** Martin A. Belzunce, Andrew J. Reader

**Affiliations:** ^1^ Royal National Orthopaedic Hospital Stanmore UK; ^2^ Department of Biomedical Engineering School of Biomedical Engineering and Imaging Sciences King’s College London London UK

**Keywords:** brain phantom, PET

## Abstract

**Purpose:**

To introduce a method that allows the generation of ultra high‐resolution (submillimeter) heterogeneous digital PET brain phantoms and to provide a new publicly available [ 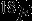 ]FDG phantom as an example.

**Method:**

The radiotracer distribution of the phantom is estimated by minimizing the Kullback–Leibler distance between the parameterized unknown phantom distribution and a radiotracer‐specific template used as a reference. The phantom is modelled using the histological and tissue classified volumes of the BigBrain atlas to provide both high resolution and heterogeneity. The Hammersmith brain atlas is also included to allow the estimation of different activity values in different anatomical regions of the brain. Using this method, a realistic [ 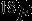 ]FDG phantom was produced, where a single real [ 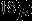 ]FDG scan was used as the reference to match. An MRI T1‐weighted image, obtained from the BigBrain atlas, and a pseudo‐CT are included to complete the dataset. A full PET‐MRI dataset was simulated and reconstructed with MR‐guided methods for the new [ 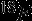 ]FDG phantom.

**Results:**

An ultra high‐resolution (400 μm voxel size) and heterogeneous phantom for [ 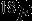 ]FDG was obtained. The radiotracer activity follows the patterns observed in the scan used as a reference. The simulated PET‐MRI dataset provided a realistic simulation that was able to be reconstructed with MR‐guided methods. By visual inspection, the reconstructed images showed similar patterns to the real data and the improvements in contrast and noise with respect to the standard MLEM reconstruction were more modest compared to simulations done with a simpler phantom, which was created from the same MRI image used to assist the reconstruction.

**Conclusions:**

A method to create high‐resolution heterogeneous digital brain phantoms for different PET radiotracers has been presented and successfully employed to create a new publicly available [ 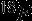 ]FDG phantom. The generated phantom is of high resolution, is heterogeneous, and simulates the uptake of the radiotracer in the different regions of the brain.

## 1 INTRODUCTION

In positron emission tomography (PET), image reconstruction methods are usually evaluated with simulated datasets generated from digital phantoms. Using simulated data is advantageous because it provides a controlled environment where a ground truth is available and therefore different quantitative image quality metrics can be computed. For example, the bias and variance in specific regions of interests (ROIs) can be used to assess image quality or performance of different image reconstruction methods.^1^, ^2^, ^3^, ^4^ In order to obtain more relevant quantitative metrics, the simulated datasets need to be as realistic as possible.

A realistic PET digital phantom is expected to be high resolution and heterogeneous over the body region represented by the phantom. It needs to be high resolution so as to be able to be affected by partial volume effects (PVEs)^5^ in the simulation process. Heterogeneity is required to account for the nonhomogeneous uptake of the radiotracer that is observed in molecular imaging.^6^ Moreover, the activity distribution depends on the molecular target of the radiotracer, therefore, a digital phantom needs to be radiotracer dependent. For example, in brain imaging, a fluorodeoxyglucose ([ 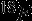 ]FDG) PET phantom should not only account for the differences in the uptake in the gray and white matter but also for the heterogeneity observed in different brain regions within these two tissue classes. Another important aspect that is frequently overlooked when analysing reconstruction methods is the evaluation of specific clinical tasks where these methods are expected to be beneficial. In the particular case of PET brain imaging, the radiotracer uptake in specific anatomical regions is of importance in the study of neurodegenerative diseases or neuroreceptors.^7^, ^8^ Accordingly, brain phantoms would ideally need to account for variations in the radiotracer uptake for each given application.

However, to the best of our knowledge all the available digital PET brain phantoms either (a) have a limited spatial resolution (making them not ideal to evaluate quantification errors due to PVE),^9^, ^10^, ^11^ (b) are not heterogeneous^9^, ^10^, ^12^ or, (c) do not take into account task‐specific scenarios.^9^, ^10^, ^12^ In addition, they are commonly piecewise constant and generated from segmented magnetic resonance imaging (MRI) images of the brain,^9^, ^11^, ^12^ therefore, quantitative errors can be underestimated when doing regularized reconstructions, especially when anatomical information is used as guidance.^13^ The latter have attracted new attention since the introduction of clinical simultaneous PET‐MRI scanners. Despite showing promising results,^13^, ^14^, ^15^, ^16^ the evaluation of MR‐guided reconstruction methods are not completely satisfactory due to possible mismatches between the MRI anatomical image and the PET radiotracer distribution.^17^ In the simulation of PET‐MRI datasets, the distribution of the PET tracer is usually generated from an anatomical image, such as a T1‐weighted MRI image, and as a consequence the PET digital phantom boundaries match perfectly the structure of the anatomical images used to assist the reconstruction (except for the introduction of small mismatches such as tumours). These limitations in the design of currently available phantoms and their respective simulated datasets mean that assessing the level of benefit of MR‐guided image reconstruction for real data is not straightforward. Specifically, the results for simulated data usually demonstrate impressive partial volume correction (PVC) and noise reduction, whereas this level of improvement is often reduced for real data.^15^, ^16^, ^18^

In this work, we present a method to generate high resolution and heterogeneous digital brain phantoms that can emulate different PET radiotracers. The method is based on the BigBrain atlas dataset,^19^ a free and publicly available ultra high‐resolution three‐dimensional model of a human brain at nearly cellular resolution of 20 μm. The activity distribution of the phantom is estimated from a template or scan used as a reference. Using the proposed method, we created an instance of an ultra high‐resolution heterogeneous PET‐MRI PET [ 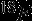 ]FDG phantom, which is publicly available. Finally, we evaluated the performance of this phantom by simulating a PET‐MRI dataset and carrying out standard and MR‐guided reconstructions.

## 2 MATERIALS AND METHODS

**Figure 1:**
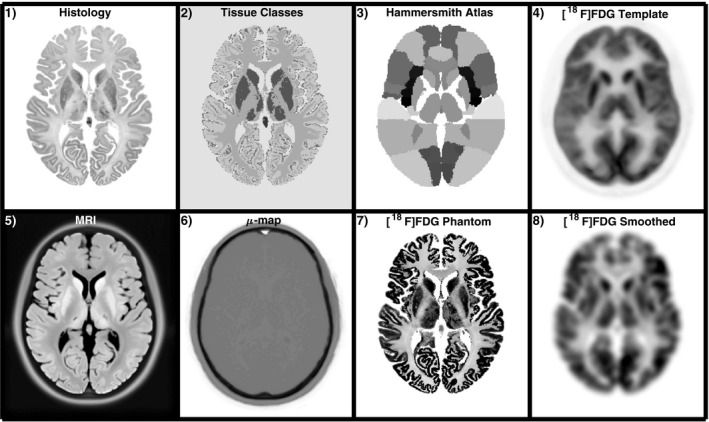
First row: datasets used in the generation of the phantom. From left to right, the histology and classified tissue images of the BigBrain atlas, the Hammersmith atlas, and a normalized[ 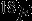 ]FDG scan used as template. Bottom row: full output ${[}^{18}F$ ]FDG PET‐MRI dataset. MRI image, attenuation map (*μ*‐map), ${[}^{18}F$ ]FDG phantom, and a smoothed version.

We propose a method to create high‐resolution heterogeneous brain phantoms using the BigBrain atlas,^19^ where histological volumes (reconstructed from 7404 histological sections) normalized into the Montreal Neurological Institute (MNI) space are available in isotropic voxel sizes that range from 100 to 400 μm. The histology images provide information of cell body density in the brain. Additionally, a classified volume with eight basic tissue classes of the BigBrain atlas was also used, where each voxel is classified into one of the following tissue classes: cortical gray matter, white matter, cerebellum, cortical layer 1, subcortical gray matter, pineal gland, cerebellum/brainstem gray and white matter. The histology and classified images are shown in the first row of Fig. 1. An MRI T1‐weighted image of the BigBrain atlas prior to being histologically processed is also available, but with inverse contrast and not normalized into MNI space.

In order to produce heterogeneous and radiotracer‐specific phantoms, the uptake in different regions of brain is estimated by using a brain atlas and a template of the radiotracer of interest, which can be a simple individual scan. In this work, we used the Hammersmith brain atlas^20^ and a reconstructed image of real data from an [ 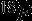 ]FDG study from a Siemens mMR scanner (used as a [ 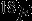 ]FDG template) to produce a realistic [ 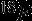 ]FDG phantom. The [ 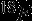 ]FDG scan corresponds to a female 17‐yr‐old epilepsy patient.

### 2.1 Generation of radiotracer‐specific phantoms

The radiotracer distribution of the phantom is estimated by minimizing the Kullback–Leibler distance between the unknown parameterized phantom distribution and a template for a given radiotracer, which is equivalent to maximizing the log likelihood for Poisson data. The latter has been widely used in the context of PET image reconstruction and it can be formulated with:

(1) $$\hat{\theta }=\underset{\theta }{\text{argmax}}\left\{L\left(\theta |m\right)-\beta U\left(\theta \right)\right\}$$

where *θ* is a vector with the parameters for the unknown phantom activity distribution and *m* is a template or single scan to be used as a reference (i.e., a [ 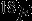 ]FDG template). In our method, the phantom activity is parameterized in a way that *θ* is a vector with the relative uptake value for every one of the 95 regions available in the Hammersmith atlas for every tissue type in the classified image of the BigBrain atlas. The Hammersmith atlas provides spatial information for the different anatomical regions in the brain (e.g., right middle frontal gyrus) while the classified image of the BigBrain atlas identifies the tissue type (e.g., gray matter), allowing the estimation of different uptake values for a given tissue type in the different anatomical regions of the brain. *βU*(*θ*) is a regularization term, which is used to penalize large differences between adjacent regions in the vector *θ*. The hyperparameter *β* controls how much these differences are penalized. Regularization is needed since this is an ill‐posed problem where there are many possible high‐resolution phantom distributions that, when resolution degraded, can accord with the low‐resolution PET scan used as a template. Without regularization [*β* = 0 in Eq. (1)], the estimated uptake values in very small regions tend to rise exponentially over iterations in the maximization of *L*(*θ*|*m*), generating a nonrealistic uptake distribution. The proposed regularization term enables generation of phantom distributions where there are no extremely high uptake differences between adjacent regions for the same tissue type.

A novel model for the phantom activity is proposed:

(2) x=HDTAθ

where *x* is a vector with the activity values of every voxel in the phantom obtained from the set of 760 parameters in vector *θ* (95 regions by 8 tissue classes). *A* is a matrix that has as many rows as voxels in the phantom and as many columns as uptake parameters to be estimated. The elements $a_{\mathrm{ij}}$ of matrix *A* give the probability that a voxel $x_{i}$ belongs to the atlas region involved in the uptake parameter $\theta_{j}$ . In this work, we use the probability maps of the Hammersmith atlas instead of the maximum probability atlas in order to avoid sharp edges between atlas regions. When using a probability map for each region, each column *j* of matrix *A* represents an image with the probability map for the region involved in parameter *j*. Because in *θ* each region of the atlas is estimated for every tissue type, in matrix *A* the probability map for each region is repeated in as many columns as tissue types are used.

*T* is a matrix that models the tissue class for each voxel, therefore, it has as many columns as voxels in the phantom and as many rows as parameters to be estimated. In practice, the matrix *TA* is computed directly instead of computing each matrix individually and then multiplying them. *D* is a diagonal matrix with the cell body density for each voxel of the phantom. It provides heterogeneity at a lower scale, based on biological information. Finally, the matrix *H* models the resolution in the template used as a reference.

**Figure 2:**
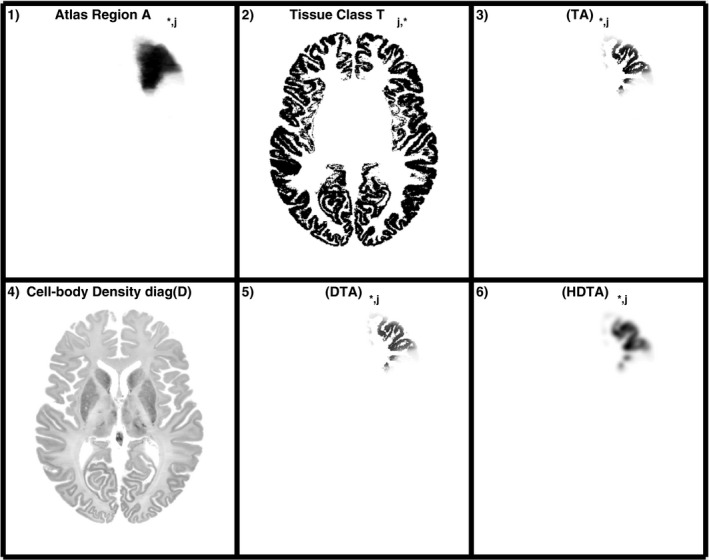
Example of one of the basis functions *j* used in the forward projection of the parameters *θ*. In this case, $\theta_{j}$ represents the uptake in the gray matter of the right middle front gyrus. The probability map of the middle front gyrus in the Hammersmith atlas (1) is multiplied by the gray matter mask from the classified image of the BigBrain atlas (2) to generate the column *j* of matrix *TA* (3). The latter is then multiplied by the diagonal matrix *D* with the cell body density of the BigBrain atlas (4) to generate the column *j* of matrix *DTA* (5). Finally, the image in (5) is smoothed to match the resolution of the template used as a reference to form column *j* of matrix *HDTA*. Images 1, 2, 3, 5, and 6 show one slice of the 3D image (which is stored as a vector in the column *j* of each matrix), while image 4 shows the same slice for the 3D image stored in the diagonal elements of matrix D.

In order to give an insight on the components of the model and their implementation, in Fig. 2 the steps performed to apply the forward model is shown for one of the uptake parameters in *θ*. In this example, $\theta_{j}$ is the uptake value for gray matter in the right middle frontal gyrus. The image in Fig. 2‐5) is one of the basis functions used in the generation of the phantom, while in Fig. 2‐6) the basis function is smoothed to match the resolution of the template.

The objective function in Eq. (1) is optimized using the one step late (OSL) MAP‐EM algorithm.^21^

### 2.2 Regularization

A quadratic regularization was used to penalize large differences in the uptake values in neighbor brain regions:

(3) $$U\left(\theta \right)=\sum_{j}^{N}\sum_{k\in N_{j}}\xi_{\mathrm{jk}}{\left(\theta_{j}-\theta_{k}\right)}^{2}$$

where for each parameter $\theta_{j}$ (which represents the uptake in the region *j* of the phantom), the distance between its centroid and the centroid of all the other regions *k* that belong to the same tissue type are calculated. The weight $\xi_{\mathrm{jk}}$ is the inverse of the Euclidean distance between centroids of regions *j* and *k*, normalized by the sum of all the inverse distances. In this way, only the uptake values $\theta_{k}$ in the regions closer to region *j* have impact on the regularization of parameter $\theta_{j}$ .

### 2.3 An [ ]FDG phantom

An instance of an [ 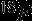 ]FDG PET Phantom was created using the method described in the previous section. A single [ 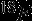 ]FDG scan was used as the template to match. The full phantom dataset was completed with MRI and computed tomography (CT) images in order to be able to simulate a PET‐MRI dataset. The MRI T1‐weighted image available in the BigBrain atlas was registered to the phantom using the symmetric normalization method (SyN).^22^ In addition, a skull obtained from a template MRI was added to the T1‐weighted image of the brain. Finally, a pseudo‐CT and its respective attenuation map was generated from the MRI image using the pseudo‐CT synthesis tool by Burgos et al.^23^ Finally, an attenuation map for 511 keV was generated from the CT image.

The proposed method was implemented in MATLAB (version R2018a) and executed on a workstation with a Dual Intel Xeon E5‐2697 processor and 256 GB of RAM.

### 2.4 Simulated data of the proposed phantom

**Figure 3:**
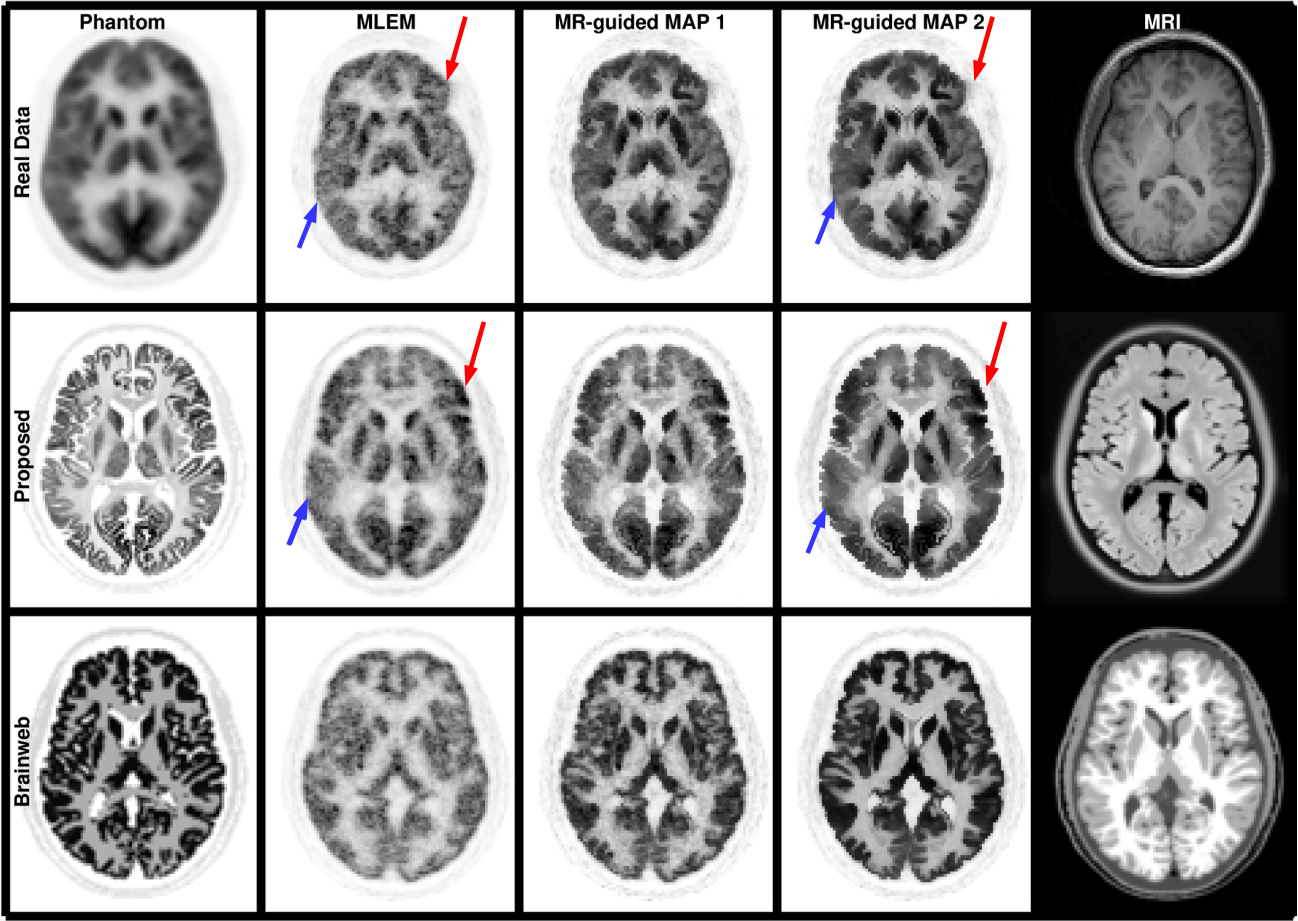
Reconstructed images for the patient data that was used as a template, and for simulated datasets using the proposed phantom and a BrainWeb phantom. The data were reconstructed with the MLEM algorithm (second column) and performing MR‐guided MAP reconstructions with mild ( $\beta \;=\;4\;\times \;10^{2}$ , third column) and strong ( $\beta \;=\;2\;\times \;10^{3}$ , fourth column) regularization. In the first column the patient data are normalized into MNI space as it was used for the estimation of the phantom activity. In the last column, the MRI of each dataset is shown. Red arrows show the higher uptake in the left frontal lobe in the patient data and phantom. Blue arrows show lower uptake in the right temporal lobe. [Color figure can be viewed at wileyonlinelibrary.com]

A brain scan was simulated using the high‐resolution heterogeneous [ 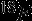 ]FDG brain phantom with a voxel size of 0.4 × 0.4 × 0.4 mm 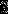 . The simulations were carried out as described in Ref. ^18^, where the resolution, the normalization factors and the geometry of the Siemens mMR PET‐MRI scanner were modelled. In this simulation framework, attenuation, randoms, and scatters effects are also taken into account. For the attenuation, the attenuation map of the proposed phantom was used (Fig. 1, 2, 3, 4–6). To simulate the PET resolution, a 4.3‐mm FWHM kernel was used, which corresponds to the spatial resolution of the Siemens mMR PET‐MRI scanner in the centre of the FOV and it is adequate for brain imaging where the point spread function of the system can be well approximated as shift invariant.^24^

**Figure 4:**
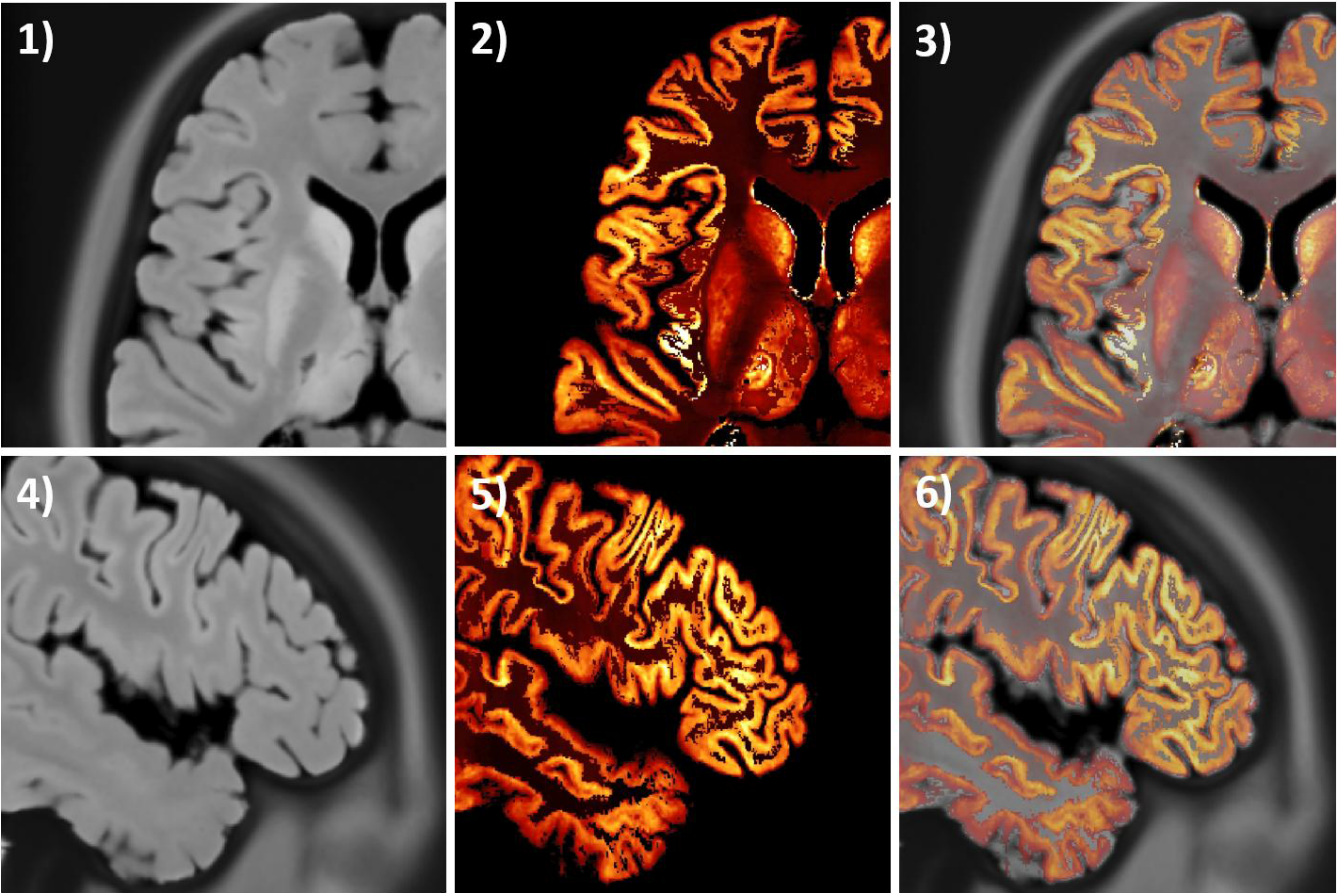
Registration of the magnetic resonance imaging (MRI) image in histology space to the phantom. In (1) and (4) transverse and sagittal planes of the MRI image. Same planes of the [ 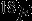 ]FDG phantom in images (2) and (5). Overlay images in (3) and (6). [Color figure can be viewed at wileyonlinelibrary.com]

The simulated data were reconstructed with MLEM^25^ and MR‐guided MAP algorithms. For the latter, we used a smoothed Lange prior with similarity weights obtained with the Bowsher method as implemented in Ref. ^18^, which were computed from the T1‐weighted image of the phantom using the 40 most similar neighbours in a 5 × 5 × 5 neighbourhood.

The same process was repeated with a brain phantom based on the BrainWeb dataset^9^ in order to draw a comparison with the proposed phantom. The BrainWeb dataset was selected for this comparison as it has been widely used to simulate PET and PET‐MRI brain scans.^13^, ^16^ The contrast between gray matter and white matter was 4:1.

## 3 RESULTS

The full phantom dataset for the [ 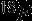 ]FDG Phantom can be seen in the second row of Fig. 1 and the dataset has been made publicly available at http://doi.org/10.5281/zenodo.1190598.

In Fig. 3, the [ 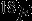 ]FDG scan used as a template is shown in the first row after being normalized into MNI space along with its reconstructed images in its original space for MLEM and MR‐guided reconstructions. The new [ 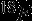 ]FDG Phantom and the reconstructed images from a simulated dataset are shown in the second row. In the third row, an [ 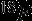 ]FDG phantom based on the BrainWeb database is shown in order to draw a comparison between both phantoms. It can be seen that the proposed phantom accounts for the heterogeneities in the [ 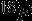 ]FDG uptake. For example, in the real data and the proposed phantom there is a higher gray matter uptake in the left frontal lobe (red arrow) and a lower uptake in the right temporal lobe (blue arrow), while for the standard BigBrain phantom the gray matter has a uniform uptake. Furthermore, the MR‐guided reconstructions of the proposed phantom obtain a more modest PVC than for the BrainWeb phantom, which accords well with the results obtained for the real data. On the contrary, the PVC performance of guided reconstructions for the BrainWeb phantom is extremely good, and therefore not realistic, due to the unrealistically perfect match between the MRI and PET boundaries (as the PET phantom was created from the MRI image).

## 4 DISCUSSION AND CONCLUSION

A method to create ultra high‐resolution heterogeneous digital phantoms is presented in this work. The method takes advantage of the publicly available BigBrain dataset to generate a PET phantom from ultra high‐resolution histology images, and in this way overcomes the problems of piecewise constant and lower resolution phantoms generated from MRI images. In this work, we employ the method to generate an [ 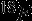 ]FDG phantom that includes all the components needed to perform PET‐CT and PET‐MRI simulations.

The method allows the generation of a wide number of different phantoms by using either a real scan or a template as a reference. Here we presented just one of the many possible instances of the phantoms that can be generated. We used an [ 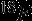 ]FDG scan of a young patient in order to generate a radiotracer‐specific phantom with heterogeneous uptake values.

In terms of the attenuation map, we used a pseudo‐CT generated with the Burgos method^23^ as it has been shown to be one of the most accurate attenuation correction techniques for PET‐MRI brain imaging^26^ and it is publicly available.

### 4.1 Limitations

A potential limitation of the [ 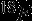 ]FDG phantom presented in this work is that the uptake distribution is estimated from a low resolution PET scan, although this would always be the case as PET images have relatively low resolution. The proposed model for the phantom and the method to estimate its distribution were designed to handle this issue. However, as there are many phantom distributions that would accord with the low‐resolution reference, the generated distribution could be suboptimal and this may be the reason why we observed a relatively low contrast in the [ 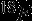 ]FDG phantom.

A second limitation of the proposed method is that there are some small mismatches between the histology images of the BigBrain and the MRI image. This could be a consequence of a suboptimal image registration in the preparation of this dataset since the MRI is not aligned to the histology images in the original BigBrain dataset. In Fig. 4, the registered MRI, the [ 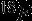 ]FDG phantom and an overlay image are shown, where a very good agreement (with just some small differences) is observed. However, these small differences could be due to the PVE as the BigBrain atlas has higher resolution than the MRI.

It could be argued that histology images with cell body density are not appropriate for an [ 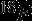 ]FDG phantom as cell bodies are not directly related to glucose metabolism.^27^ This limitation, only in effect at a very small scale, is compensated for by using an atlas and a template of the radiotracer of interest to estimate the uptake in different regions of the brain. In this way, an [ 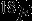 ]FDG phantom with a realistic radiotracer distribution (out of many possible distributions) is obtained.

### 4.2 Conclusions

In this work, we propose a novel method to generate digital brain phantoms, which we employed to create an [ 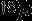 ]FDG phantom. The complete dataset, including the example [ 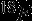 ]FDG phantom, the pseudo‐CT, the *μ*‐map, and the T1‐weighted images, is available online. The generated phantom fulfils the requirements of being ultra high resolution (i.e., voxel sizes down to 100 μm), by being heterogeneous at a small scale (heterogeneity provided by the cell body concentration in the histology images), not being generated from a segmented MRI image, and also being heterogeneous at a larger scale to simulate the uptake of a given radiotracer.
